# Oxidative Stress and Mitochondrial Dysfunction in Human Diseases: Pathophysiology, Predictive Biomarkers, Therapeutic

**DOI:** 10.3390/biom10111558

**Published:** 2020-11-16

**Authors:** Chia-Jung Li

**Affiliations:** 1 Department of Obstetrics and Gynecology, Kaohsiung Veterans General Hospital, Kaohsiung 813, Taiwan; nigel6761@gmail.com; Tel.: +886-7-342-2121 (ext. 71562); 2 Institute of BioPharmaceutical Sciences, National Sun Yat-sen University, Kaohsiung 804, Taiwan

Mitochondria are important sites for a variety of cellular processes, including amino acid and fatty acid metabolism, the citric acid cycle, nitrogen metabolism, and oxidative phosphorylation to produce ATP. During the production of ATP, the waste produced by mitochondria is called free radicals. This toxic waste can cause specific changes (mutations) in the genetic material of the mitochondria that damage the mitochondrion itself and can cause cell dysfunction and disease. Mitochondrial disease occurs when the production of cellular energy is defective. Because of the omnipresence of ROS in cells and contribution of mitochondria in the production and removal of cellular ROS, a greater understanding of oxidative stress in mitochondria, under both normal and disease-causing conditions, and the involvement of mitochondrial ROS in the global regulation of gene expression, can illuminate the contribution of mitochondria in the development of disease and may lead to the advancement of new and novel therapeutic modalities that exploit mitochondria in treating many maladies. 

The scope of this Special Issue is to provide a broad and updated overview on oxidative stress and mitochondrial dysfunction in human disease. The collection includes five original research papers and one review article from well-known experts in the field and provides the interested reader with specific examples of mitochondrial dysfunctions in human disease. Importantly, the articles both highlight the most recent results and illustrate perspectives for future research. This Special Issue thus furthers our understanding of mitochondrial medicine, accumulated in the past, and presents challenges that will need to be addressed by the scientific community in the near future.

The study by Nambo-Venegas et al. [1] explored the spinocerebellar ataxia type 7 (SCA7), a neurodegenerative disease characterized by cerebellar ataxia and retinal degeneration, and mainly caused by the abnormal CAG repeat amplification in the coding region of the ATXN7 gene. The onset and severity of SCA7 vary greatly between patients, so there is a need to identify sensitive biomarkers that can accurately diagnose the disease and monitor its progression. The authors performed metabolomics analysis of acyl carnitine, free carnitine and amino acids in plasma samples of SCA7 patients. They identified promising metabolites that could serve as auxiliary biomarkers for the diagnosis and prognosis of the disease because they are sampled in a relatively non-invasive manner and are readily detected by easy biochemical assays that could be implemented in clinical laboratories.

The article by Zalewska et al. [2] studied the relationship between ceramides, the mitochondrial respiratory system, oxidative stress, inflammation, and apoptosis in the submandibular gland mitochondria of mice with insulin resistance. Their study pointed out that feeding mice a high-fat diet not only induces systemic insulin resistance, but also is accompanied by higher activity of pro-oxidant enzymes and reduced mitochondrial complex function. Furthermore, the high-fat diet impairs the redox homeostasis of saliva, and leads to oxidative damage and increased apoptosis of submandibular mitochondria.

The article by Badshah et al. [3] proved the hypothesis that caffeine regulates oxidative stress, neuroinflammation and synaptic dysfunction in the brains of mice injected with LPS. The protective effect of caffeine on oxidative damage caused by LPS is induced by increasing the level of Nrf2/HO-1 and reducing the performance of TLR4/NF-kB/JNK, thereby greatly reducing cell apoptosis. This shows that caffeine reduces oxidative stress, neuroinflammation and synaptic dysfunction in the brains of LPS-injected mice. 

The article by Chen et al. [4] studied the effect of resveratrol (RSV) on mitochondrial activity and fibrosis in mice infected with *Schistosoma japonicum*. RSV leads to increased protein expression of *Δψm* and PGC-1α (related to mitochondrial biogenesis), thereby improving the function of mitochondria in the liver of mice infected with *Schistosoma japonicum*. RSV treatment also resulted in a decrease in the degree of liver fibrosis, as indicated by the decreased protein expression of the fibrosis markers collagen I, collagen III, and αSMA. As a final effect, RSV causes a decrease in the number of worms in the liver, and also damages the surface and internal tissues of *Schistosoma japonicum* worms in mice infected with *Schistosoma japonicum*. The findings indicate that RSV may have immense potential as a direct or indirect therapeutic agent for the treatment of schistosomiasis.

The article by Huang et al. [5] explored the protective effect of Hispidin, a group of polyphenols isolated from Phellinus igniarius, on oxidative stress induced by hydrogen peroxide in ARPE-19 cells. They proposed a new function of hispidin, which protects ARPE-19 cells from oxidative damage induced by H2O2 by inhibiting the level of ROS. The results show that hispidin induces Nrf2/HO-1 expression through the JNK-Nrf2-dependent pathway in ARPE-19 cells, thereby inhibiting H2O2-induced oxidative stress and cell death. This also shows that hispidin has the potential to be a candidate therapeutic drug for the treatment or prevention of age-related macular degeneration.

The paper by Bryll et al. [6] extensively reviewed the currently reported role and mutual interactions of oxidative damage and impaired glucose metabolism as key factors affecting metabolic complications in schizophrenia. These observations may be a premise for novel potential therapeutic targets that will delay not only the onset of first symptoms but also the progression of schizophrenia and its complications.

This Special Issue describes important findings related to mitochondrial dysfunction, and dysregulation in several diseases, such as neurodegenerative disease, insulin resistance, neuroinflammation, schistosomiasis, age-related macular degeneration, and schizophrenia, and also highlights the most recent progress in the knowledge and the clinical and pharmacological applications related to the most relevant areas of healthcare. This may be helpful in assessing prognostic or predictive indicators, as well as developing new therapies and new insights aimed at improving human disease.
